# The Essex-Lopresti lesion

**DOI:** 10.1007/s11751-012-0149-0

**Published:** 2012-10-25

**Authors:** K. Wegmann, J. Dargel, K. J. Burkhart, G. P. Brüggemann, L. P. Müller

**Affiliations:** 1 Klinik und Poliklinik für Orthopädie und Unfallchirurgie, Universitsklinikum Köln, Kerpener Straße 62, 50937 Cologne, Germany; 2 Institute of Biomechanics and Orthopaedics, German Sport University Cologne, Am Sportpark Müngersdorf 6, 50933 Cologne, Germany

**Keywords:** Essex-Lopresti lesion, Longitudinal forearm instability, Radial head fracture, Distal radio-ulnar joint, Interosseous membrane, Radial head prosthesis

## Abstract

The Essex-Lopresti lesion represents a severe injury of the forearm unit. In the 1940s, it’s pathology and consequences have already been mentioned by several authors. Over the course of time, the pathophysiology of the lesion was displayed in more detail. Therefore, an intensive analysis of the involved anatomic structures was done. The interosseous membrane was shown to play a major role in stabilising the forearm unit, in the situation of a fractured radial head, which is the primary stabiliser of the longitudinal forearm stability. Moreover, biomechanical analyses showed a relevant attribution of the distal radio-ulnar joint to the forearm stability. If, in the case of a full-blown Essex-Lopresti lesion, the radial head, the interosseous membrane and the distal radio-ulnar joint are injured, proximalisation of the radius will take place and will come along with secondary symptoms at the elbow joint and the wrist. According to actual studies, the lesion seems to occur more often than realised up to now. Thus, to avoid missing the complex injury, subtle clinical diagnosis combined with adequate imaging has to be undertaken. If the lesion is confirmed, several operative treatment options are available, yet not proofed to be sufficient.

## Introduction

The human forearm is an essential working unit in daily life. Its enormous capacity of use is not solely facilitated by the movement in the wrist and elbow but also involves pivotal pronation and supination. With this combination of movements, the forearm forms a determining tool in human efficiency. The ability to pronate and supinate the forearm represents a deciding step forward in human processing [4]. In addition, injuries to the forearm can have a relevant impact on the wounded individual. In addition to Galeazzi fracture, Monteggia fractures complete forearm fractures and solitary radial head fractures, the Essex-Lopresti injury is a condition of dramatic changes to the structures and the function of the forearm following axial trauma [22, 29, 36, 59]. This combination of radial head fracture, lesions of the interosseous membrane (IM) and lesions of the distal radio-ulnar joint (DRUJ) was first clinically described by Curr and Coe in 1946 [13]. However, the fracture combination was named after the British surgeon Peter Essex-Lopresti, who presented two cases in 1951 [22]. Essex-Lopresti recognised the relevance of the instability inherent in the complex lesion. He described proximalisation of the radius, which was worsened by radial head excision, as well as ulno-carpal impingement and radial deviation of the wrist [22]. He advised early recognition of the lesion to prevent chronic changes to the elbow and wrist. In 1992, Trousdale et al. [84] published a series of 20 patients treated for Essex-Lopresti lesions. Trousdale made clear that the misdiagnosed and delayed treated Essex-Lopresti injuries clearly exhibit worse clinical outcomes. Like Essex-Lopresti, Trousdale found the instability to be the cause of the torturing symptoms of pain at the elbow and wrist and deformation of the forearm [84]. Several clinical and biomechanical studies have followed [6, 63, 64, 68, 76], but no definitive treatment strategy has yet been established for acute or chronic lesions, and poor clinical outcomes remain common [15, 45, 60, 64, 72, 78, 81, 83]. Furthermore, the rate of undiagnosed Essex-Lopresti injuries may be high, as the study of Trousdale et al. [84] implies. Only 25 % of patients with Essex-Lopresti injuries in their review were initially correctly diagnosed. Edwards and Jupiter [18] also pointed to the high risk of underdiagnosing the Essex-Lopresti lesions on first presentation. In a recent MRI study of acute low grade radial head fractures, Hausmann [32] identified involvement of the IM in 9 out of 14 patients. Duckworth et al. [17] identified radial shortening of 2–4 mm, suggestive of an Essex-Lopresti lesion, in 9 % of their 60 total patients. Thus, accompanying lesions of the stabilising ligamentous structures must be considered in radial head fractures, which account for approximately 30 % of bony lesions to the elbow and 5 % of all fractures [40]. Nonetheless, the literature has thus far referred to the Essex-Lopresti lesion as a rare entity, found in approximately 1 % of all radial head fractures [22, 84].

In this article, we provide further understanding of the stability of the forearm by an insight into its anatomy, illustrate the most likely mechanism of trauma of Essex-Lopresti injuries and review the treatment options for cases of acute and chronic instability.

## Anatomy of forearm stability and biomechanics

Linked together via the proximal (PRUJ) and distal (DRUJ) radio-ulnar joint and connected by the IM, the human forearm works as a dynamic unit, contributing to the extraordinary mobility of the human upper limb. The forearm is subjected to axial, rotational and transverse forces [67]. In vivo, the most relevant forces are axial loads in the proximal direction. Under these conditions, the main stabiliser, referred to as the primary stabiliser, of the forearm is the radial head, articulating at the PRUJ with the radial notch of the proximal ulna. If articulating with an intact capitulum, there is no proximalisation of the radius [68]. The PRUJ is mainly stabilised by its bony configuration and the annular ligament, which keeps the radial head in the radial notch [44]. The secondary forearm stabilisers include the DRUJ and the IM. The DRUJ is a much more complex joint than the PRUJ [30]. Due to its relatively incongruous bony components, which make a great range of motion possible, the DRUJ depends on tenuous ligamentous support. Within the DRUJ, the dorsal and palmar radio-ulnar ligaments, the ulno-carpal ligaments, the distal portion of the IM and the TFCC work as passive stabilisers. The pronator quadratus was identified as an active stabiliser that functions by pressing the ulnar head and the sigmoid notch together [30]. Also, the extensor carpi ulnaris (ECU) tendon and sheath have been shown to play an important role in DRUG stability [2, 26].

**Fig. 1:**
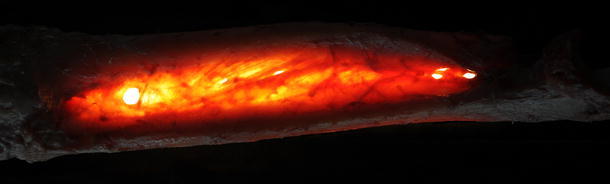
Dissected forearm unit in backlight photography. Illustration of the different fibre bundles of the interosseous membrane

**Fig. 2:**
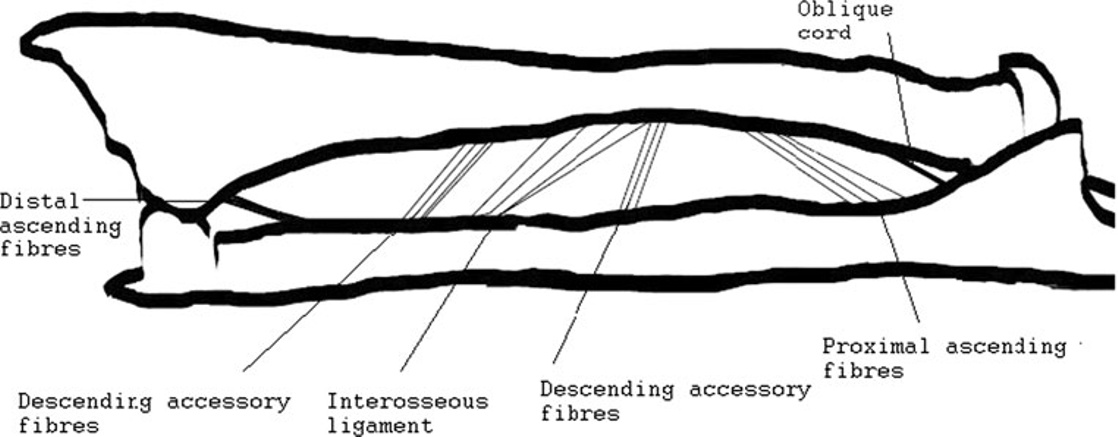
Illustration of the ascending and descending fibres of the interosseous membrane

The IM has recently been characterised as a complex, partly membranous and partly ligamentous structure and has been linked to several critical roles in forearm function [3, 36, 67, 68, 75]. The IM can be distinguished into two layers of fibres, as so called anterior and a posterior division, which travel from the radius to the ulna (Figs. 1, 2). The anterior division, which can be further divided into three groups, is comprised of descending fibres, including a noticeable central portion of fibres [67, 68]. Skahen et al. [75] refer to these central fibres as the central band, whereas Hotchkiss et al. [36] advocated referring to it as the interosseous ligament (IL) due to its ligamentous structure and biomechanical behaviour. The IL is found at the mid-portion of the anterior division at 62 % of the radial shaft length, starting from the styloid process [56]. The IL exhibits an average width of 2.6 cm and travels distally from the radius to the ulna at an approximate angle of 21° to the ulnar axis [75]. Several authors have characterised the IL as the major portion of the IM responsible for stabilising against proximalisation of the radius [36, 67]. The dorsal division can be further subdivided into two groups consisting of fibres ascending from the radius to the ulna. By their ascending orientation from the radius to the ulna, the fibres are largely responsible for preventing distalisation of the radius [67].

With its different subdivisions, the intact IM works together with the DRUJ as a secondary forearm stabiliser. Hotchkiss et al. [36] demonstrated that the IL accounts for 71 % of the overall stiffness of the IM experienced after resection of the radial head. The DRUJ adds 8 % to the overall stiffness after radial head resection. Moreover, Hotchkiss et al. identified the IM to be the most important restraint against proximal migration of the radius in the case of a fractured or resected radial head.

The IM also plays an important role in transferring loads from the hand to the elbow. Several authors demonstrate that 80 % of an axially applied load is taken up by the distal radius at wrist level, while 20 % of the load is supported by the distal ulna. Close to the elbow joint, the load distribution decreases to 60 % on the radial and 40 % on the ulnar column [6, 31]. This distribution changes dramatically after resection of the radial head, as shown in cadaveric studies and as discussed in the following chapter [74].

## Trauma mechanisms of Essex-Lopresti lesions and the biomechanics of the instable forearm

In 1951, Essex-Lopresti pictured “a violent longitudinal compression” to the forearm as the mechanism of trauma responsible for the fracture of the radius with distal radio-ulnar dislocation [22]. Today, the cause is still believed to be the application an axial load to a pronated forearm, as during a fall [11, 15, 29]. In 2003, McGinley et al. [55] demonstrated that the pattern of injury by an axial load depends on the rotational position of the forearm; with the forearm pronated, the resultant injury may be a fracture of the radial head and lesions to the IM.

**Fig. 3:**
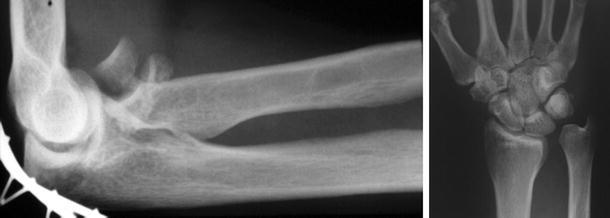
Comminuted fracture of the radial head. Resulting in proximalisation of the radius and dislocation of the unstable DRUJ

In the full-blown Essex-Lopresti lesion with fracture of the radial head, rupture of the IM and lesions of the DRUJ, the critical primary and secondary forearm stabilisers are not possible. Due to the longitudinal instability, axial loading results in the radius being pushed proximally. Birbeck et al. [6] confirmed these pathomechanics in cadaver studies. They observed no load shift between the radius and ulna in forearms with an intact radial head but dissected IM and TFCC, and of the majority of the load travelled along the radius. If combined with a compromised radial head, that is, a comminuted fracture, or after resection of the radial head, this will lead to significant proximalisation and radio-capitellar impingement [68, 74, 84]. Hotchkiss et al. [68] measured up to 7 mm of proximalisation of the radius in cadaveric forearms with fractured radial heads. In forearms with additional lesions to the secondary stabilisers, considerably more proximal migration took place [36]. At the wrist, radial shortening will result in positive ulnar variance with concomitant ulno-carpal impaction (Fig. 3). According to Shepard et al. [74], the load in the distal ulna increases by 10 % with every millimetre of proximal radial migration. In the clinical setting, this increasing load may result in the accelerated degeneration of the wrist.

Skahen et al. [75] demonstrated a relevant increase in strain in the IL of the IM after resection of the radial head and artificial loading. This result might be an explanation for late proximal radial migration in patients after radial head excision [27, 58].

## Clinical presentation and diagnosis

The clinical course of acute and chronic Essex-Lopresti lesions with forearm instability must be distinguished. In the case of an acute Essex-Lopresti lesion caused, for example, by a fall on the outstretched arm, the patient will present with significant pain over the elbow joint due to the fracture of the radial head. There will be distinct pain on palpation over the radial head. Moreover, limited elbow motion may be observed. Asymptomatic forearms and wrists in Essex-Lopresti lesions at first presentation have been encountered [22, 29]. Therefore, it is all the more important that a thorough clinical examination including the careful palpation of the forearm and the DRUJ be conducted. There can but does not necessarily have to be pain or swelling over the forearm and wrist. Tenderness over the forearm on palpation can be a sign of involvement of IM. If acute longitudinal instability is present, there can be prominence of the distal ulna at the dorsal aspect of the wrist. Pain during palpation of the DRUJ will also be a sign of involvement of the distal stabilisers [87]. DRUJ stability can be evaluated by moving the distal ulna palmar and dorsally against a fixed radius. Relevant displacement of the ulna in relation to the uninjured side and pain over the DRUJ during manipulation suggests ligament injury [46].

In chronic cases, the clinical symptoms are dictated by the above-mentioned proximal migration of the radius with radio-capitellar impingement and ulno-carpal impaction by positive ulnar variance. The history of the patients will reveal a precedent trauma to the forearm with severe radial head fracture or even radial head resection. In addition, the patients might present with increasing pain over the wrist and elbow, paired with decreasing range of motion over time. Reduced grip-strength is often observed as well. On examination, there will be painful restriction of pronosupination as well as extension and flexion at the wrist and elbow. In ulnar positive variance, the ulno-carpal impaction decreases supination and extension over the course of proximal radial migration. This is due to a dorsal and, in relation to the radius, more distal position of the distal ulna, which conflicts with the carpus during motion [34, 35].

On plain X-rays of the elbow, the fracture of the radial head should be obvious. If concomitant injury is suspected, plain X-rays of the wrist and forearm serve as a basic diagnostic method. Epner defined the optimal way to image the DRUJ and a possible ulnar variance. He advocates a p.a. roentgenogram, the shoulder abducted to 90°, the elbow flexed to 90° and the forearm in a neutral position [19]. To identify dynamic instability at the wrist, a dorso-palmar X-ray of the injured side in pronation under full grip-load might elicit hidden ulnar positive variance [39]. In chronic cases, signs of degenerative disease at the ulno-carpal or radio-capitellar region can be observed. Clearly, proximal migration of the radius and disruption of the DRUJ on first sight is not necessarily encountered, as illustrated by Rodriguez-Martin [69]. For further diagnostics, ultrasonography has been shown to offer high sensitivity and specificity in diagnosing disruption of the interosseous membrane. The intact IM presents in ultrasonography as a hyperechoic structure whose continuity or discontinuity is verifiable [23, 38]. If concomitant soft tissue lesions are suspected along with radial head fractures MRI scans in addition to X-rays are advocated. In cadaveric studies, the efficacy of MRI scans has been proven with high sensitivity and specificity [24, 57]. Furthermore, Hausmann [32] as well as Starch et al. [79] was able to distinguish acute lesions to the IM in vivo with MRI scans. However, there are no studies concerning the efficacy of MRI in revealing chronic IM lesions. Confirming this, Stevenson et al. [80] recently reported a case of a misleading MRI scan in a patient having suffered an Essex-Lopresti lesion. The patient had been treated with radial head prosthesis to possibly facilitate healing of the ruptured IM. To reassure adequate healing, an MRI scan was performed, revealing a positive result. Nonetheless, after removal of the implant, the patient developed severe symptoms due to proximalisation of the radius. Thus, MRI scans of the IM should always to be considered with care.

To be used as an intraoperative diagnostic tool, Smith et al. [76] recommend the radius-pull test to reveal longitudinal instability in forearms. With a force of 90 N, the radius is pulled proximally. The ulnar variance at the wrist is measured by fluoroscopy. If the radius is observed to migrate proximally more than 3 mm, injury to the IM is suspected. Proximal migration of more than 6 mm indicates severe impairment of all soft tissue stabilisers. More recently, Soubeyrand et al. [77] presented the radius joystick test, claiming to have found a simple intraoperative test to diagnose the disruption of the IM. To perform the test, after exposing the proximal radius, it is grasped with forceps and pulled laterally in maximum pronation. If pivoting of the proximal radius around its distal end is encountered during the manoeuvre, the forearm is positive for IM injury. The test’s promising applications must be validated in a larger series.

## Therapeutic options

**Fig. 4:**
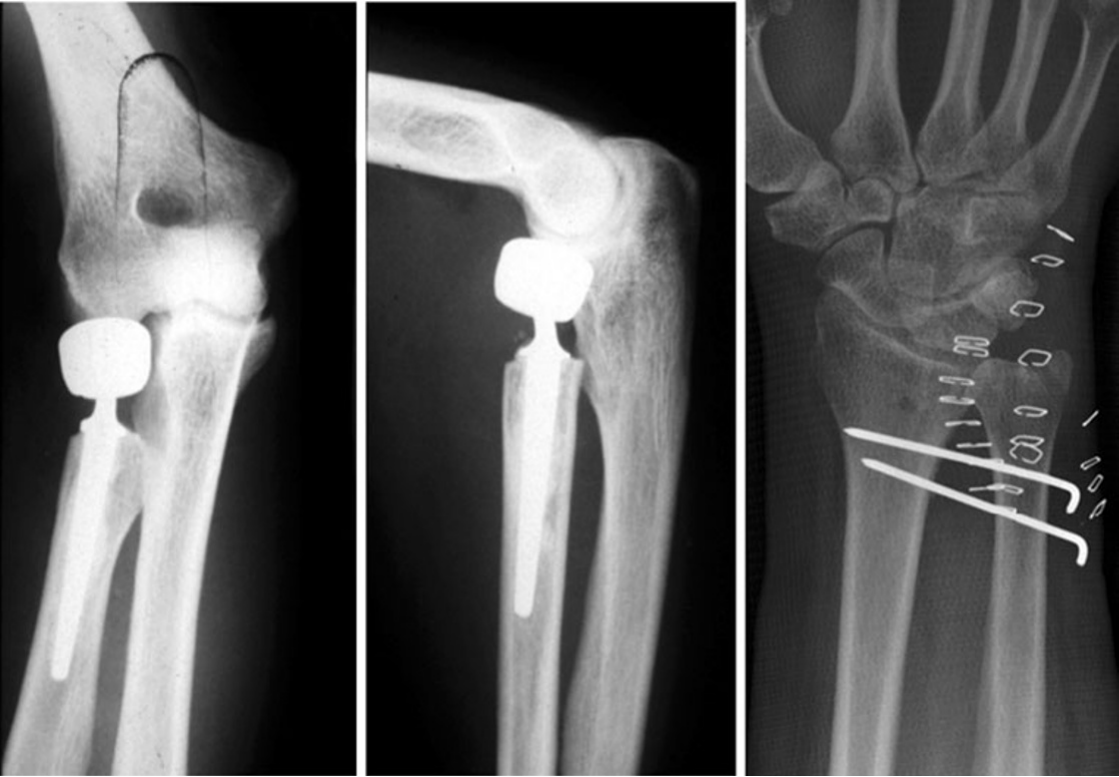
Forearm unit after metallic radial head prosthesis and temporary K-wire fixation of the DRUJ

In acute lesions of the radial head and the stabilising soft tissues, restoration of the longitudinal stability is critical to prevent proximal migration of the radius [29, 63]. Thus, only resection of the radial head is not recommended. Essex-Lopresti realised in 1951 that resection of the radial head can result in fatal sequelae for the forearm unit [22]. Even in cases with stable IM and DRUJ, proximal radial migration can take place, probably due to attenuation of the IM and other soft tissue stabilisers [18, 75]. Trousdale et al. [84] reported 20 patients with radio-ulnar dissociation, of whom 15 were diagnosed correctly with a mean delay of 7 years. All of those 15 patients had been treated at some point with radial head resection. According to their elbow and wrist scores, only 12 of the 15 patients exhibited satisfactory results. Thus, preserving the radial head might be beneficial, even in the case of intact secondary stabilisers. The fractures of the radial head commonly are categorised by the Mason classification into Types I through IV [52]. Type I refers to non-displaced fractures that are typically treated conservatively. Type II fractures are minimally displaced and are treated with osteosynthesis [33, 47]. Type III is comprised of displaced and comminuted fractures, whereas Type IV fractures represent radial head fractures with accompanying disruption of the collateral ligaments in the course of elbow dislocation. For these comminuted fractures, several treatment protocols exist. The most favourable of these options is the reconstruction of the forearm. If reconstruction is not possible, replacement should be conducted. The sole resection of the radial head, as is recommended by some authors, should be avoided. Proximal migration of the radius, concomitant positive ulnar variance and the possible sequelae represent serious consequences of radial head resection. Accordingly, authors reported poor results after only resecting the radial head [21, 34]. Advanced implant material with mini-fragment internal fixation sets and anatomic plates is available [8, 9]. Koslowsky et al. [49] presented a method in 2007 of internal fixation of comminuted radial head fractures with the newly designed FF-system (Orthofix, Germany). In a prospective study, 23 patients with Types III and IV radial head fractures achieved promising results. However, internal fixation of the radial head is a feasible but, nonetheless, challenging surgical procedure. The surgeon, as well as the patient, should be prepared for the case of an irreparable fractured radial head and inevitable resection. In such cases of comminuted radial head fracture where internal fixation is not possible, replacement has been established. Allograft, metal and silicone implants are available (Fig. 4). Karlstad et al. [45] reported the failure of fresh frozen allografts in treating longitudinal instability with radial head replacement. Thus, the authors advocated stepping back from allograft replacement. Similarly, the desired clinical outcome could also not be achieved with silicone implants. Mayhall et al. [53] reported the failure of silicone implants. Inflammatory arthritis has been observed in elbow joints after radial head replacement with silicone prostheses [86], and several other authors confirmed poor clinical outcomes with silicone prostheses [61, 88]. Unlike these reports, radial head replacement with metallic implants appears to exhibit better outcomes. In a series of 31 patients, Knight et al. [48] observed satisfying results with metallic radial head replacement due to complex fractures, including restoration of longitudinal stability and a low rate of dislocation. Moro et al. [60] reported good and excellent results in the Mayo performance score after metallic radial head prosthesis in 17 out of 25 patients, despite reduced grip-strength and significantly reduced flexion and extension. In 2010, Burkhart et al. [7] reported good clinical results in 17 patients treated with bipolar radial head prosthesis after a follow-up of 8.8 years. However, further long-term results in clinical studies must be conducted with regard to loosening and wear.

For the restoration of forearm stability, secondary stabilisers have to be addressed. By reconstruction of the DRUJ, one critical secondary stabiliser is approached [15, 63]. If disrupted, the repair of the TFCC has to be considered in the acute setting. Arthroscopic and open procedures have been described [14, 54, 71, 85]. In 2008, Anderson et al. [5] found no significant difference in outcomes in a clinical comparison of arthroscopic and open repair of TFCC-tears. If acute instability is due to injury to the radio-ulnar ligaments, open or arthroscopic debridement is recommended when conservative treatment has been exhausted [15]. In cases of chronic injury, however, TFCC-repair alone is not sufficient, and complex restoration of DRUJ stability with tissue reconstruction is necessary. Kakar et al. [41] described several surgical techniques dealing with chronic DRUJ instability. The technique established by Adams and Berger involves the reconstruction of the dorsal and palmar radio-ulnar ligaments in an anatomic fashion by guiding an autologous tendon graft through the origin and insertion of the radio-ulnar ligaments [1]. Using this technique, Adams and Berger as well as other authors reported promising results in treating chronic DRUJ instability [73, 82].

As mentioned above, the IM plays an important role in forearm stability. However, its reconstruction is controversial, particularly in the acute setting. Some investigators recommend acute treatment by immobilising the forearm in supination to make healing of the IM possible. This can be accomplished by splinting or even fixing the DRUJ with K-wires for 2 months [36, 62] (Fig. 4). However, the capacity of the IM for healing may be limited. Stevenson et al. [80] report a case of failed healing of the IM after 2.5 years. In addition, Gong et al. [28] reported failed healing of the IM after immobilisation with a cast and pinning. Marcotte et al. [51] suggested that forearm muscles interfere with the healing of the IM by interposition. There have been few reports of direct repair of the IM. Failla et al. [23] described the direct repair of the IM using an approach via the interval between the M. extensor digitorum communis and the M. extensor digiti quinti. The repair was performed by direct suture of the disrupted IM-tissue. On a short-term follow-up, the procedure exhibited positive clinical results in combination with radial head fixation and ORIF of the DRUJ. Nonetheless, the optimal method of treatment of acute disruptions of the IM remains to be established, and this method must be disclosed to the patient if acute treatment is mandatory. Moreover, one must ask if the immobilisation of acute injured forearms in supination is reasonable in conservative treatment. Gabriel et al. [25] proved in a biomechanical setting that strain in the IM is highest in supination. McGinley et al., Hotchkiss et al. and other authors confirmed this finding [12, 36, 55]. This observation is particularly important when assuming that ligament healing will work best if its disrupted borders are slackened [20, 37, 43]. Therefore, further development of the therapeutic options for conservative treatment is necessary. In cases of chronic IM disruption, repair is not possible. Therefore, numerous surgical procedures for the reconstruction of the IM have been proposed by several authors to date. Tendon grafts of M. palmaris longus, M. flexor carpi radialis, Achilles-tendon, bone-patellar-bone grafts, pronator teres muscle and synthetic materials have all been used [11, 42, 51, 66, 70, 72, 75]. In biomechanical cadaver studies, Pfaeffle et al. [66] achieved adequate stabilisation of the IM and restoration of normal load transfer using the flexor carpi radialis tendon in a double-bundle technique. Sellman et al. [72] gained adequate stiffness of the IM using a braided polyester cord in combination with metallic radial head replacement. Kam et al. [42] achieved sufficient radio-ulnar stability in cadaveric studies by reconstructing the IL with a suture button construct. Drake et al. [16] percutaneously placed a suture button into cadaveric forearms with cut IM and resected radial heads, thereby restoring the longitudinal stability provided by the IM. Marcotte and Osterman presented a series of 16 patients with longitudinal radio-ulnar dissociation. In a series of follow-ups averaging 78 months post-intervention, the authors observed reduced wrist pain, improved grip-strength and low complication rates. The authors combined an ulnar shortening osteotomy with the implantation of a bone-tendon-bone autograft from the patellar ligament [51]. Nonetheless, many other authors have been unable to develop stability comparable to the original physiologic status. Using a single FCR-tendon, Skahen et al. [75] were unable to restore forearm stability in cadavers after sectioning the IL and the TFCC. Similarly, the reconstruction of the IM by Stabile et al. [78] exhibited significantly inferior stiffness compared to the physiologic IM. They evaluated the stiffness of Achilles-tendon, FCR-tendon and patellar bone-tendon-bone transplants. In addition, Teiwanj et al. [81] were able to obtain only minor stability using FCR-tendon, Palmaris longus tendon and patellar bone-tendon-bone grafts. Overall, Stabile and Teiwanj report the most stable reconstruction results with bone-tendon-bone grafts, but these grafts remain significantly inferior to physiologic stability [78, 81]. To date, no sufficient treatment protocols have been established.

As the ultimate salvage procedure in the case of continuing instability, radio-ulnar synostosis has been used [50, 65]. Alternatively named one-bone-forearm, ulnar synostosis describes the fixation of the radius and ulna in a neutral or slightly pronated position. As it is regularly in use in congenital pathologic conditions of the forearm, ulnar synostosis exhibits usually disappointing clinical results in cases of traumatic genesis [10]. Thus, the procedure comes at the expense of forearm rotation, and it should be avoided as long as possible.

A synopsis of the treatment recommendations for the Essex-Lopresti injury reveals the lack of long-term results in clinical studies in larger series. Because of the rarity of full-blown forearm instability, the relevant data are difficult to locate. In addition, the biomechanics in healthy individuals are not fully understood, as is evident by the differing results in experimental series. Overall, the unsatisfying treatment results and 80 % of failure in chronic forearm instability repair [35, 70] mandate further efforts to analyse the pathologic biomechanics and develop in vitro treatment methods [45, 84]. Last, loading patterns in the forearm after the reconstruction of forearm stability remain to be studied.
